# A case report of integrating Chinese and Western medicine: A new era in the treatment of stiff person syndrome

**DOI:** 10.1097/MD.0000000000036883

**Published:** 2024-01-12

**Authors:** Xiaohui Lu, Du Hong, Wenshuo Wu, Liping Zhang, Changlin Qiu

**Affiliations:** a Zhejiang University of Chinese Medicine, Hangzhou, Zhejiang Province, China; b Zhejiang Provincial Hospital of Chinese Medicine, Hangzhou, Zhejiang Province, China.

**Keywords:** case report, Chinese medicine, stiff person syndrome (SPS), TCM

## Abstract

**Rationale:**

At present, there are limitations to the treatment of stiff person syndrome (SPS). Current treatments are still ineffective or financially burdensome for some patients, so it is imperative to explore more appropriate treatments for patients. This is a case report of a SPS with a more significant effect of combined Chinese and Western medicine, which may provide new treatment ideas for other patients.

**Patient concerns:**

This patient presented with episodes of stiffness and pain in the lower back and lower extremities. His electromyography shows continued activation of normal motor units in the paraspinal and abdominal muscles. However, relevant laboratory tests including glutamic acid decarboxylase antibody and Amphiphysin antibody were negative. After a period of treatment including clonazepam, baclofen, prednisone and intravenous immunoglobulin, this patient experiences a shortened maintenance period of medication, accompanied by symptoms such as emotional anxiety and cognitive decline, which severely affects his life.

**Diagnoses:**

This patient was diagnosed with SPS.

**Interventions:**

In May 2022 the patient decided to combine Chinese medicine for simultaneous treatment.

**Outcomes:**

During the period of simultaneous treatment with Chinese and Western medicine, the patient experienced remission of clinical symptoms, reduction of concomitant symptoms and improved quality of life.

**conclusion:**

A combination of Western and Chinese medicine was effective in relieving this patient pain and stiffness and reducing the patient anxiety. Combined Chinese and Western medicine treatment may be able to bring better results to more patients with stiff person syndrome.

## 1. Introduction

### 1.1. Overview

Stiff person syndrome (SPS) is a rare autoimmune neurological disorder that presents with muscle stiffness and trigger-induced painful muscle spasms, mainly affecting the proximal and axial muscles of the limbs.^[1]^ The current estimate of the annual incidence of SPS is about one in a million, with the age of onset mostly between 20 and 50 years and a higher rate in women than men.^[2,3]^

The pathogenesis of SPS is unclear, and it is currently thought that glutamic acid decarboxylase (GAD) is involved in the synthesis of the inhibitory neurotransmitter gamma-aminobutyric acid (GABA). Exposure of GAD antigen during synaptic vesicle secretion induces the body to produce GAD antibodies, which react immunologically with GAD in the nerve endings of GABAergic neurons, resulting in reduced GABA synthesis and impaired transport, leading to persistent muscle excitation.^[4–6]^ In addition, SPS can be a sign of Paraneoplastic syndrome. Some patients have antibodies related to paraneoplastic syndrome, such as gephyrin antibody and Amphiphysin antibody, which affect inhibitory neurotransmitters such as GABA and glycine to produce the corresponding clinical symptoms.^[7]^

The diagnosis of SPS requires a comprehensive assessment, with a high suspicion of the following symptoms when approached: Muscle stiffness, prominent in the abdomen, thoracic spine and paraspinal spine. Continuous concerted muscle contraction, clinically and electrophysiologically confirmed. Paroxysmal spasms caused by unexpected noise, tactile stimuli or emotional disturbance. No other neurological disorders that could explain the stiffness and rigidity. Immunological tests suggesting positive GAD (65) antibody or Amphiphysin antibody. Benzodiazepine treatment is effective.^[4,5,8]^ The treatment of SPS is mainly symptomatic and immunotherapy, in addition to physiotherapy and interventions.^[9–13]^ Treatments are shown in Table 1.

**Table 1:** the main treatments for SPS.

Main treatment methods	Daily dose	Pharmacology	Side effects
1.Main medicines			
Diazepam	5–100 mg	*GABAA agonist	Dizziness, drowsiness, tiredness, anxiety
Clonazepam	2.5–6 mg	GABAA agonist	
Alprazolam	2–4 mg	GABAA agonist	
Baclofen	10–60 mg	†GABAB agonist	
Tizanidine	6 mg	inhibits norepinephrine release	
Levetiracetam	2000 mg	Inhibition of GABAergic transmission	Psychiatric and behavioral side effects (PBSEs)^[14]^
Pregabaline	300 mg	Structurally related to GABA but mechanism of action unknown	
Gabapentin	3600 mg		
2.Immunotherapy			
Intravenous immunoglobulin	2 g/kg in 2–5 d	Immunomodulation	Infusion related reactions (IRR)
Rituximab	375 mg/m^2^		
Tacrolimus	3 mg		
Prednisone	50–60 mg/d		
Plasma exchange (PE)	5 PE in 1–2 wk		
3.Interventional therapy			
Anesthesia	Lack of relevant literature reports		
Spinal cord Stimulation			
4.Physical Therapy			

* GABAA: gamma-aminobutyric acid type A receptor.

† GABAB: type B gamma-aminobutyric acid receptor.

SPS = stiff person syndrome.

Early treatment is considered essential for the maintenance of mobility in people with SPS. Untreated patients usually develop disability and may become increasingly difficult to treat as the disease progresses. Most patients experience relief from stiffness and spasticity after treatment. Unfortunately, there are also many patients who develop symptoms and disability despite a combination of treatments.^[2,3]^

### 1.2. Case review

**Figure 1.**
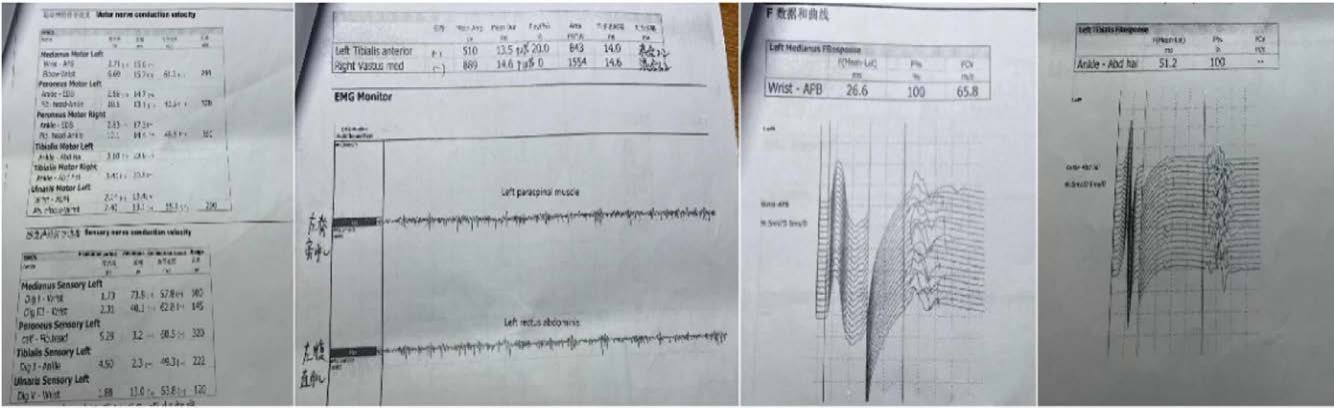
Electromyography report of the patient.

The patient is a 40-year-old male with a previous diagnosis of hyperlipidemia and a history of smoking and alcohol consumption, but family history was negative. This patient presented in September 2020 after a prolonged drive with significant back pain on walking, but relieved in the recumbent and sitting positions. He first underwent a brief magnetic resonance imaging of the lumbar spine at the local hospital, which suggested a lumbar disc herniation but no spinal cord compression. He then experienced an up-and-down extension of his back pain, an increase in the frequency of episodes and, within 6 months, episodes of lower limb stiffness and spasms with stooping and unsteady gait, a condition that was easily precipitated by emotional stress. The patient was seen at Peking Union Medical College Hospital in July 2021. During the physical examination the patient walked with anterior trunk flexion and a widened step base. Muscle tension was present when the physician palpated the psoas major, abdominal muscles and groin area. The rest of the physical examination was unremarkable. Further laboratory tests on the patient revealed negative serum and cerebrospinal fluid for GAD antibodies, amphiregulin antibodies, cerebrospinal fluid routine, cerebrospinal fluid biochemical parameters and oligoclonal bands. But the electromyography showed continuous activation of normal motor units of the paraspinal and abdominal muscles. The patient electromyography report is shown in Figure 1. The final diagnosis of SPS was made in conjunction with the patient symptoms, signs and relevant laboratory investigations.

The course of Western medical treatment for this patient was as follows: Intravenous immunoglobulin (0.4g/kg, 8 September—September 12, 2021), Oral prednisone 60 mg Quaque die supplemented with clonazepam (morning—noon—evening—bedtime, 2 mg—2 mg—2 mg—3 mg), baclofen 15 mg Ter in die. On September 14, 2021 the patient was discharged from the hospital and treated according to prednisolone acetate 60 mg, reduced by 5 m every fortnight to 10 mg maintenance, clonazepam and baclofen dosage as in hospital. He continued to have pain and stiffness in his abdomen and lower limbs after the above treatment. As of May 3,2022, clonazepam had been increased to 4 mg 4 times daily, baclofen to 20 mg 3 times daily and methylprednisolone 10 mg once daily, but the patient still felt that the treatment was not satisfactory. This unsatisfactory efficacy is mainly evidenced by the fact that patients continue to have recurrent episodes of unbearable low back pain and the frequency of low back pain and spasm attacks remains high after treatment with the basic drug dose. Symptoms can be relieved for a short time after raising the dose of drugs, but after 1 to 2 weeks there is a decrease in effectiveness. In addition, he often suffers from intolerable side effects such as memory loss, fatigue and emotional stress after the dose has been increased. He was first treated with traditional Chinese medicine (TCM) synchronization on May 3, 2022, when the western dosage was clonazepam 4 mg 4 times daily, baclofen 20 mg 3 times daily and prednisolone acetate 10 mg once daily, along with regular intravenous immunoglobulin.

### 1.3. Chinese medicine intervention process

The patient was started on May 3, 2022 on a combination of Chinese and Western medical treatment. At the time, this patient was experiencing frequent episodes of low back pain, which could be triggered by emotional stress or after sitting for long periods of time. In addition to these main symptoms, he also suffered from anxiety, memory problems, sleep disturbances, a feeling of weakness and urinary disturbances. Based on the patient back pain, easy sweating, poor sleep, fear of heat, emotional anxiety, a bit of purple dusky tongue, coarse sublingual veins, lightly greasy coating, and thin and string pulse, the pattern of the disease are liver-kidney yin deficiency and phlegm stasis. Therefore, Chinese Medicine used enrich and nourish the liver and kidney, dispel wind and unblock the collaterals, dry dampness and dissolve phlegm as the basic treatment methods, and formulated herbal prescriptions suitable for this patient according to the treatment methods. Decoct the Chinese herbal drink that adjusts the patient constitution and relieves the patient symptoms for half an hour to obtain 200 mL of the drug mixture to be consumed twice a day (at 8 am and 4 pm). See Table 2 for the prescription of the herbal composition.

**Table 2:** Chinese herbal medicine prescription.

Classification in traditional Chinese medicine	Compositions in Chinese name	Compositions in Latin name	Daily dosage	Modern pharmacology
Tonifying liver and kidney medicine	Dihuang	Rehmannia glutinosa	15 g	Anti-fatigue,^[15]^ Neuroprotective,^[16]^ anti-aging effects,^[17]^ etc
	Wuzhuyu	Tetradium ruticarpum	12 g	Regulating central nervous system (CNS) homeostasis, antitumor,^[17]^ etc
	Niuxi	Achyranthes bidentata Blume	12–20 g	Immnopotentiation, Promoting nerve growth and preventing neuronal apoptosis^[18]^
	Wuweizi	Fructus Schisandrae chinensis	6 g	Antioxidation, suppression of apoptosis, anti-inflammation, regulation of neurotransmitters, and modulation of brain-derived neurotrophic factor (BDNF)^[19]^
“Huatan Chushi” medicine	Dannanxing	Arisaema cum Bile	9 g	Anti-inflammatory and analgesic, anti-epileptic, antioxidant,^[20]^ etc
	Banxia	Pinellia ternata	9 g	Antitussive, expectorant, antiemetic, antitumor, antibacterial, and sedative-hypnotic activities^[21]^
“YangXueQuFeng” medicine	Shaoyao	Paeonia lactiflora Pall	15–20 g	Anti-Inflammatory, Analgesic, Immunomodulatory effects,^[22]^ etc
	Gegen	Pueraria lobata	15 g	antioxidative and immunoregulatory effects,^[23]^ etc
“Qufeng Tongluo” medicine	Chantui	Cicadae Periostracum	9 g	Anticonvulsive, sedative and hypothermic effects^[24]^
	Jiangcan	Bombyx batryticatus	15 g	Anticonvulsant, antiepileptic, hypnotic and neurotrophic effects^[25]^
	QuanXie	Scorpion	6 g	Analgesic, Anti-epileptic, Influences neurotransmitter release,^[26]^ etc
	Dilong	Pheretima	6 g	Contains peptides with analgesic and anti-inflammatory activity. Inhibitory effect on fibroblast cells^[27]^
	Qishe	Pheretima	9 g	Analgesic effects^[28]^
	Fengfang	Vespae nidus	5 g	Immunomodulatory effects^[29]^
Medication during follow-up consultations				
Tonifying liver and kidney medicine	Duzhong	Eucommia ulmoides	15 g	Antioxidant, anti-inflammatory, neuroprotective, anti-fatigue, anti-aging, anti-cancer and immunoregulation activities,^[30]^ etc
	Sangjisheng	Viscum coloratum	15 g	Antiseptic, emetic, purgative, anti-inflammatory, anti-arrhythmic, antispasmodic, anti-psychotic, and anti-epileptic properties^[31]^
	Congrong	Cistanche salsa	15 g	Its purified product, echinacoside has neuroprotective effects and prevent liver injuries^[32]^
	Xuduan	Dipsacus asper Wall	12 g	Anti-osteoporosis, neuroprotective, anti-uterine contraction, hepatoprotective, anti-myocardial infarction, anti-inflammatory and anti-arthritis^[33]^ and so on.
	Gouji	Rhizoma cibotii	15 g	Analgesic, anti-cancer, hemostatic,^[34]^ etc
“Huatan Chushi” medicine	Peilan	Eupatorium fortunei	9 g	Anti-inflammatory and regulating glucose and lipid metabolism through multiple components^[35]^
	Caodoukou	Semen Alpiniae Katsumadai	9 g	Stomachic, antiemetic, inhibition of tumor formation, antioxidant, anti-inflammatory and antibacterial^[36]^
	Houpo	Magnoliae Officinalis Cortex	9 g	ntibacterial, anti-tumor, analgesic, anti-inflammatory and anti-oxidative effects^[37]^
	Xiangfu	Cyperus rotundus L	9 g	Antioxidant, anti-allergic, antipyretic, anti-inflammatory, antiemetic, hepatoprotective, anti-diarrhea, anti-malarial, neuroprotective,^[38]^ and so on.
“Wen li” medicine	Wuyao	Lindera aggregata	10 g	Anti-hyperlipidemic, anti-tumor, anti-inflammatory, analgesic, and anti-oxidant^[39]^
	Yizhiren	Alpinia oxyphylla Miq	12 g	Ameliorating cognitive disorders and alleviating pathological brain injuries characteristic of neurodegenerative disorders^[40]^
“Xingqi Zhitong” medicine	Chuanlianzi	Toosendan Fructus	12 g	Anti-tumor, anti-inflammatory, analgesic, antiviral, anti-oxidative, insecticidal, antifeedant and other properties^[41]^
	Foshou	Citri sarcodactylis Fructus	9 g	Analgesic, anti-inflammatory, antidepressant^[42]^
“Yangyin Shengjin” medicine	Zhimu	Anemarrhenae rhizoma	12 g	Anti-inflammatory, antioxidant, antipyretic, antidepressant^[43]^
	Shihu	Dendrobium nobile	12 g	Antioxidant, antitumor, anti-inflammatory, and immunomodulatory activities^[44]^

Through Table 2, we found that according to the patient ‘s liver and kidney deficiency, phlegm and blood stasis syndrome, Chinese medicine prescriptions mainly used tonic drugs and remove pathological factors drugs. Tonic medicine is a kind of TCM with the main functions of enhancing human activity function, improving disease resistance and eliminating weak syndromes. The tonic drugs in this prescription can be divided into liver and kidney tonic drugs, blood tonic drugs, yang tonic drugs and yin tonic drugs. Removal of pathological factors drugs refers to the drugs that can remove the pathological factors that hinder human health due to insufficient function of zang-fu organs or the influence of surrounding environment. There are mainly 3 types of drugs in the prescription: removing phlegm and dampness, promoting blood circulation and qi circulation, and dispelling wind and dredging collaterals. It is worth noting that tonic medicines and medicines to remove pathological factors are often complementary to each other. For example, blood nourishing medicines can remove wind by nourishing the blood, which in Chinese medical theory is called nourishing the blood to remove wind.

### 1.4. Results

**Figure 2.**
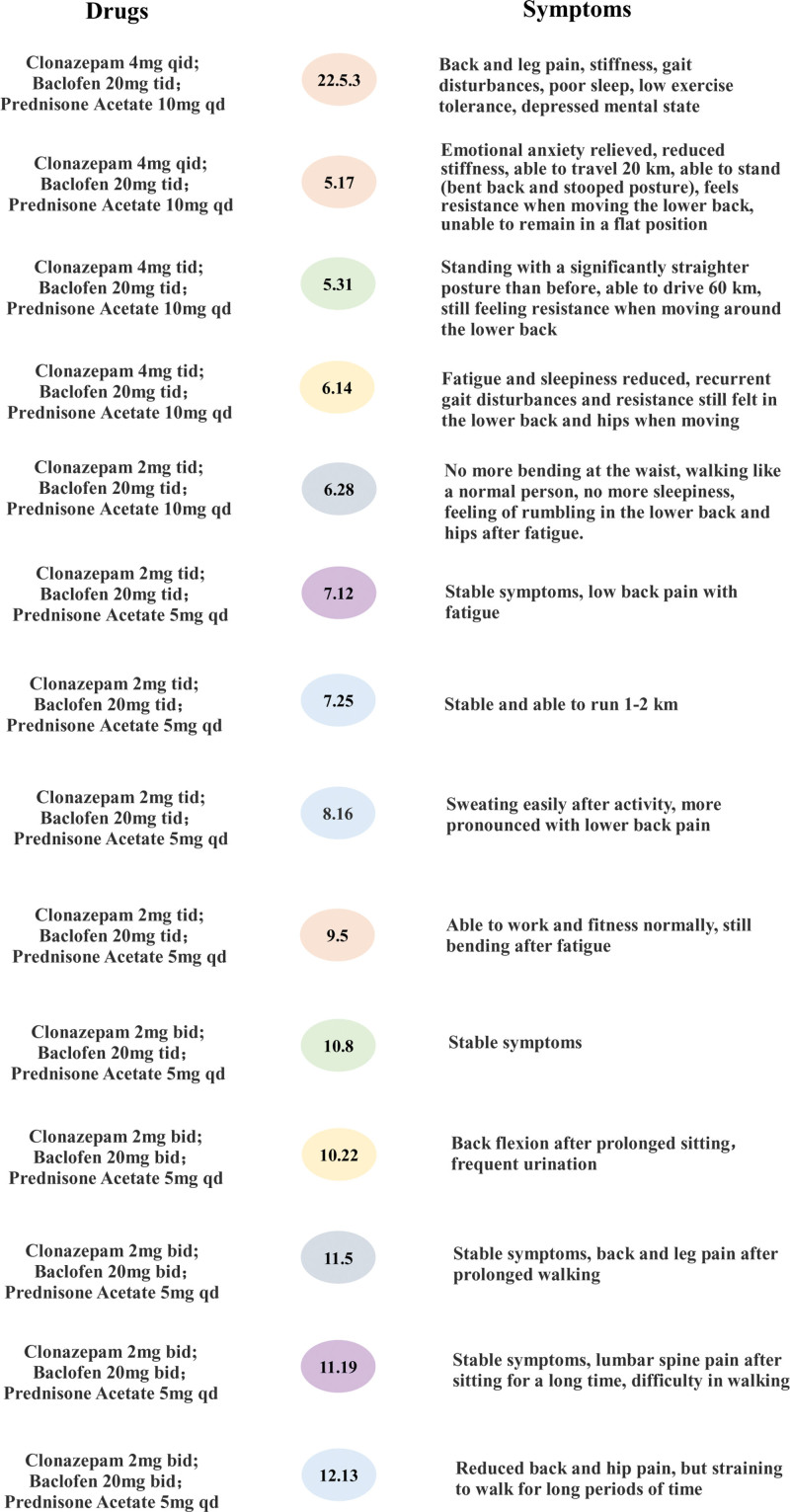
The patient consultation process.

After 2 weeks of taking the herbal preparation, the patient emotional tension and stiffness improved and he was able to drive on his own, although he experienced lower back pain after driving 20 km. After 1 month of taking the Chinese medicine preparation the patient has been able to walk upright and can drive 60 km on his own. There was no worsening of symptoms even when the dosage of clonazepam was halved. With continued combination of Western and Chinese medicine, the patient was gradually able to run and work out. And the dosage of western medicine is also gradually reducing the dosage. As of December 13, 2022, he had been treated with a combination of Chinese and Western medicine for more than 6 months, during which time his activity tolerance had increased, his back pain, muscle stiffness, and spasticity episodes had decreased, the dosage and frequency of use of Western medications had decreased, and the patient symptoms had stabilized and his condition was under control. The patient attendance is shown in Figure 2.

## 2. Discussion

SPS does not have the same name as a disease in TCM theory, but based on the main symptoms of stiffness and spasticity, SPS can correspond to the name “convulsive disease” in TCM theory. The classification of diseases in TCM is based on the patient symptoms and physical signs, and through the 4 methods of “Inspection, Inquiry, Auscultation and Olfaction, Palpation and Pulse-taking” a comprehensive assessment of the patient condition is carried out to find the pathological mechanism and formulate appropriate treatment for the patient specific condition. This process of diagnosis is referred to as “Pattern Differentiation to treat” in Chinese medical theory. According to TCM theory, the patient was diagnosed with “convulsive disease: yin deficiency of liver and kidney, phlegm-damp stasis signs.” On the one hand, the patient age, stiffness, back pain and string pulse are indications of liver-kidney yin deficiency. On the other hand, a bit of purple dusky tongue, coarse sublingual veins and lightly greasy coating can be attributed to phlegm stasis.

The complex composition of Chinese herbs produces a variety of pharmacological effects after concoction. Some studies have demonstrated that Dihuang has the ability to inhibit inflammatory response signaling pathway proteins, reduce the expression of pro-inflammatory response factors, inhibit glial cell activation and improve inflammatory damage.^[45,46]^ Shichangpu and Banxia have an effect of increasing GABA and decreasing Glutamate. In addition, Shichangpu and Banxia also promoted GABA-receptor expression, and Shichangpu also up-regulated the level of GAD65, the rate-limiting enzyme for GABA synthesis.^[47]^

The formula also contains a number of worm medicines (Quanxie, Jiangcan, Fengfang, Dilong, Qishe, and Chanyi), which in TCM theory have the effect of dispelling wind and unblocking the collaterals. Numerous clinical and pharmacological studies have proved that Quanxie and Jiangcan can increase the release of GABA, prevent damage to GABAergic interneurons and reduce the excitability of neurons. Qishe and Chanyi have reduced vascular permeability, sedative, analgesic and anticonvulsant effects^[25,48,49]^ This is the first report of the use of TCM combined with medicine in the treatment of patients with SPS. During more than 7 months of treatment the patient clinical symptoms were reduced and the dosage of western medicine was decreased, which was confirmed by the patient.

This report suggests that TCM is effective in the treatment of this SPS patient, especially in relieving the patient pain and stiffness. TCM may increase the effect of western medicines and decrease their side effects when combined with them. Chinese medicine is widely used in Asia as a reliable alternative medicine and is recognized by a number of people. The combination of Chinese and Western medicine may provide new treatment ideas for more patients with SPS.

### 2.1. Limitations of the report

This case report still has some limitations. Firstly, this is only an isolated case report of the combination of Chinese and Western medicine in the treatment of SPS, and a large number of prospective studies are needed to further confirm this. Secondly, although the active ingredients of herbal medicines have been identified and some pharmacological mechanisms have been proposed in previous studies, more evidence is needed to support these theories. Finally, the effectiveness of the patients’ Chinese and Western medical treatments was mainly based on the patients’ description of symptomatic improvement, which has a more subjective limitation. To overcome this problem further, pre- and post-treatment assessment scales or laboratory test results are needed.

## Author contributions

**Writing – original draft:** Xiaohui Lu, Wenshuo Wu.

**Writing – review & editing:** Du Hong, Liping Zhang, Changlin Qiu.
